# Transcriptome-Wide Map of N^6^-Methyladenosine Methylome Profiling in Human Bladder Cancer

**DOI:** 10.3389/fonc.2021.717622

**Published:** 2021-11-15

**Authors:** Aolin Li, Ying Gan, Congcong Cao, Binglei Ma, Quan Zhang, Qian Zhang, Lin Yao

**Affiliations:** ^1^ Department of Urology, Peking University First Hospital, Beijing, China; ^2^ Institute of Urology, Peking University, Beijing, China; ^3^ National Urological Cancer Center, Beijing, China; ^4^ Beijing Key Laboratory of Urogenital Diseases (Male) Molecular Diagnosis and Treatment Center, Beijing, China; ^5^ Guangdong and Shenzhen Key Laboratory of Male Reproductive Medicine and Genetics, Institute of Urology, Peking University Shenzhen Hospital, Shenzhen-Peking University-The Hong Kong University of Science and Technology Medical Center, Shenzhen, China

**Keywords:** m6A (N6-methyladenosine), MeRIP-seq, bladder cancer, mRNA, lncRNA, circRNA

## Abstract

N^6^-Methyladenosine (m^6^A) is the most widespread internal RNA modification in several species. In spite of latest advances in researching the biological roles of m^6^A, its function in the development and progression of bladder cancer remains unclear. In this study, we used MeRIPty -55-seq and RNA-seq methods to obtain a comprehensive transcriptome-wide m^6^A profiling and gene expression pattern in bladder cancer and paired normal adjacent tissues. Our findings showed that there were 2,331 hypomethylated and 3,819 hypermethylated mRNAs, 32 hypomethylated and 105 hypermethylated lncRNAs, and 15 hypomethylated and 238 hypermethylated circRNAs in bladder cancer tissues compared to adjacent normal tissues. Furthermore, m^6^A is most often harbored in the coding sequence (CDS), with some near the start and stop codons between two groups. Functional enrichment analysis revealed that differentially methylated mRNAs, lncRNAs, and circRNAs were mostly enriched in transcriptional misregulation in cancer and TNF signaling pathway. We also found that different m^6^A methylation levels of gene might regulate its expression. In summary, our results for the first time provide an m^6^A landscape of human bladder cancer, which expand the understanding of m^6^A modifications and uncover the regulation of mRNAs, lncRNAs, and circRNAs through m^6^A modification in bladder cancer.

## Introduction

Bladder transitional cell carcinoma (TCC) is the most common urothelial tumor in urology departments in China. The vast majority originated from epithelial tissue, and TCC accounts for more than 90% (1–3). At present, the diagnosis of bladder transitional cell carcinoma mainly relies on invasive cystoscopy and pathological biopsy. The biggest difficulty in the treatment of bladder cancer is its easy recurrence. Early detection of bladder cancer can improve the chances of bladder preservation and overall survival. After bladder-sparing tumor resection, even with regular infusion of chemotherapy into the bladder, there is still a 10% to 40% recurrence rate, and some of them also show grade, stage progression, or metastasis (4, 5). Recent studies have shown that the occurrence and development of urothelial carcinoma of the bladder are closely related to changes in DNA methylation levels (6). A great deal of research has been done on the pathogenesis of bladder cancer, and numerous pathways and mechanisms involved in the progression of bladder cancer have been discovered, such as proto-oncogene activation, tumor-suppressor gene inactivation (point mutation, rearrangement, deletion), and chromosomal abnormalities (7, 8). However, many molecular mechanisms involved in the development and progression of bladder cancer remain unclear. Therefore, clarifying the molecular mechanism of the occurrence and progression of bladder cancer provides an experimental basis for the discovery of new molecular biological markers of bladder cancer and has important significance and application value for improving the survival rate of patients with bladder cancer.

More than 100 types of RNA modifications have been confirmed in mammalian cells, among which N^6^-methyladenosine methylation modification is the most common in mRNA and non-coding RNA (9). In recent years, the application of transcriptomic MeRIP-seq technology and the confirmation of m^6^A demethyltransferase and methyltransferase complex have provided a new sight for the study of the biological function of m^6^A, as well as the diversity of biological functions regulated by them. It is proved that m^6^A is a dynamic and reversible RNA modification mode (10–12). In the nucleus of cells, the m^6^A modification of mRNA is dynamically catalyzed by the methyltransferases METTL3 and METTLl4, as well as the demethyltransferase FTO and ALKBH5 (13). MeRIP-seq revealed that m^6^A methylation modification was widely distributed in the transcription region, and there was about one m^6^A modification site in every 2,000 base pairs. There are about 12,000 m^6^A loci in more than 7,000 human genes, with an average of one to three loci in each transcript, which exist in the conserved sequence RRACH (R=A, G; H=A, C or U), and mostly located near the stop codon, 3′-UTR, and long exon of transcript (14, 15).

Transcriptome refers to the collection of all RNA that is transcribed in a specific tissue or cell at a certain developmental stage or functional state, including protein-coding mRNA and non-coding RNA (16, 17). A large number of studies have shown that m^6^A methylation modification is involved in the regulation of RNA processing, growth and development of the body, the occurrence of diseases, and other physiological and pathological processes. In addition, it also plays an important role in the occurrence and development of leukemia, malignant glioma, lung cancer, liver cancer, breast cancer, and other malignant tumors (18–21). These studies have shown that abnormal mRNA and non-coding RNA epigenetic modification leads to abnormal oncogene expression, and there may be an internal relationship between m^6^A methylation and malignant transformation of cells. However, the exact mechanism and its role in tumorigenesis have not been clarified. In this study, we used MeRIP-seq and RNA-seq to research the difference of mRNA, lncRNA, and circRNA expression levels and m^6^A methylation levels between bladder cancer tissues and normal adjacent tissues. This proved that abnormal m^6^A methylation modifications in bladder cancer might directly modulate gene expression. Finally, we hope this study will facilitate further investigations of potential roles of m^6^A modification in bladder cancer pathogenesis.

## Results

### General Features of m^6^A Methylation Modification in Bladder Cancer Tissues and Tumor-Adjacent Normal Tissues

**Figure 1:**
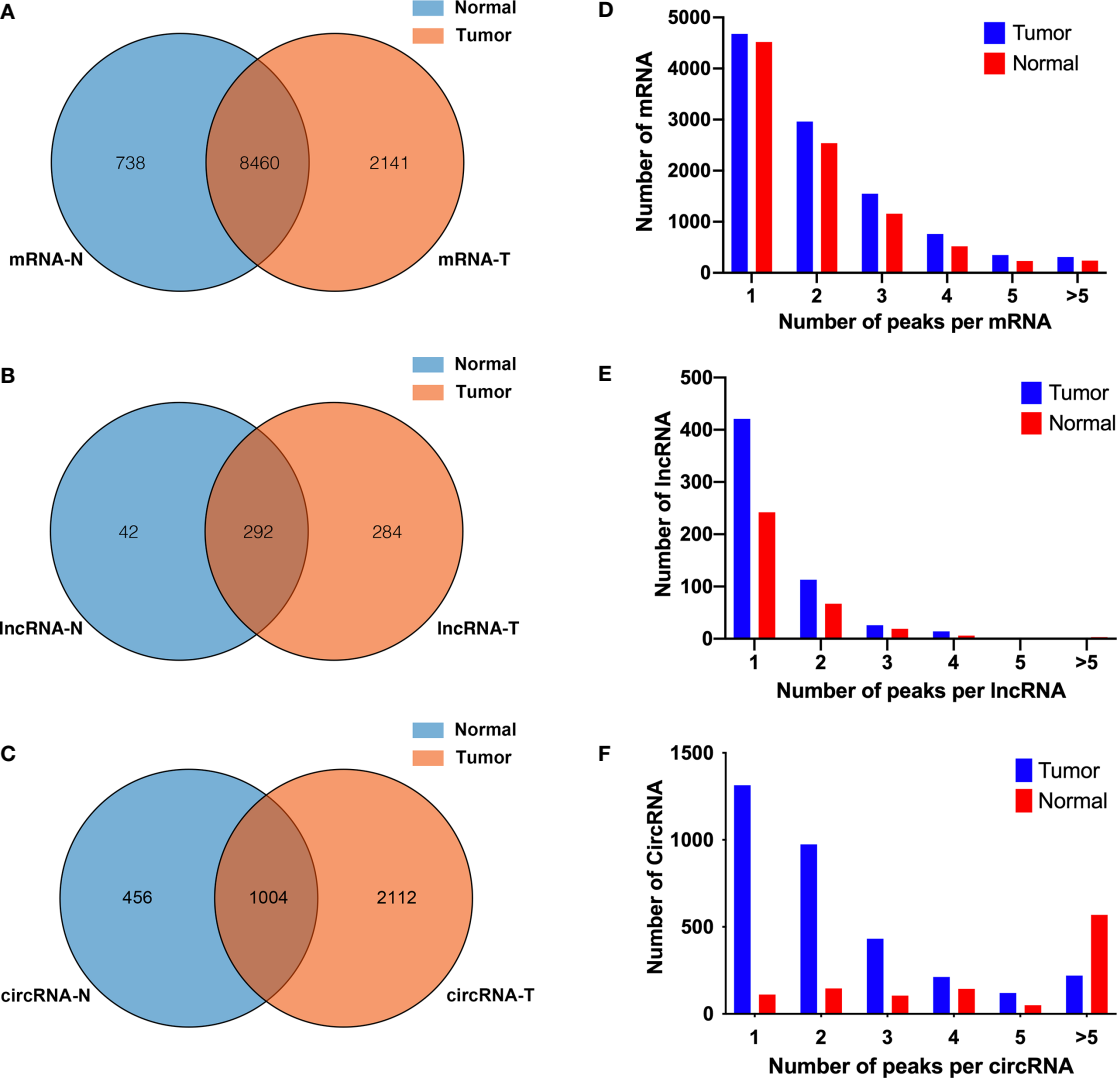
Overview of N^6^-methyladenosine methylation within mRNAs, lncRNAs, and circRNAs in bladder cancer tissues and adjacent normal tissues. **(A–C)** Venn diagram showing the overlapped m6A peaks within mRNAs **(A)**, lncRNAs **(B)**, and circRNAs **(C)** between the two groups. **(D–F)** Proportion of mRNAs **(D)**, lncRNAs **(E)**, and circRNAs **(F)** harboring different numbers of m^6^A peaks in two groups.

Human bladder cancer tissues and tumor-adjacent normal tissues from five patients were used for MeRIP-seq analysis. In tumor tissues, we detected a total of 10,601 m^6^A peaks within mRNAs (**Figure 1A**), 576 m^6^A peaks within lncRNAs (**Figure 1B**), and 3,116 m^6^A peaks within circRNAs (**Figure 1C**). While in adjacent normal tissues, there were a total of 9,198 m^6^A peaks within mRNAs (**Figure 1A**), 334 m^6^A peaks within lncRNAs (**Figure 1B**), and 1460 m^6^A peaks within circRNAs (**Figure 1C**). Among them, 8,460 m^6^A peaks within mRNAs (**Figure 1A**), 292 m^6^A peaks within lncRNAs (**Figure 1B**), and 1,004 m^6^A peaks within circRNAs (**Figure 1C**) were overlapped between adjacent normal and tumor tissues and shown by a Venn diagram. Compared with normal tissues, 4,537 new peaks appeared in tumor tissues, and 1,236 peaks disappeared, indicating that the global m^6^A modification patterns were significantly different between two groups (**Figures 1A–C**). We then examined the distribution of m^6^A methylation modifications in the human transcriptome. We found that most of methylated sequences within mRNA, lncRNA, and circRNA in adjacent normal and tumor tissues contained less than five m^6^A peaks, while few of them contained five or more sites (**Figures 1D–F**). The top 10 hypermethylated and hypomethylated m^6^A-modified peaks for bladder cancer tissues are listed in **Tables 1**, **2**.

**Table 1:** The top 10 hypermethylated m6A-modified peaks for bladder cancer tissues compared to normal tissues.

Chromosome	txStart	txEnd	Gene name	Fold change
5	17217588	17275945	BASP1	2100
19	13153029	13154610	IER2	2060
4	169991056	170001971	MFAP3L	1660
4	140622112	140624180	TBC1D9	1560
14	37591089	37594046	FOXA1	1510
9	87706143	87707673	DAPK1	1420
10	71750316	71755453	VSIR	1410
14	61279395	61281482	TMEM30B	1340
5	179863143	179865408	TBC1D9B	1260
6	21593722	21597186	SOX4	1210

**Table 2:** The top 10 hypomethylated m6A-modified peaks for bladder cancer tissues compared to normal tissues.

Chromosome	txStart	txEnd	Gene name	Fold change
2	27080260	27086378	EMILIN1	1590
11	77666800	77676890	RSF1	1260
8	93733433	93737331	RBM12B	1230
1	34855325	34856648	SMIM12	1190
10	122210373	122215428	TACC2	957
2	226794992	226799759	IRS1	891
13	26046145	26050763	SHISA2	820
2	202036201	202036860	FZD7	806
19	23323997	23324475	AC010300.1	790
8	143915176	143926881	PLEC	787

### Distribution of m^6^A Modification in Bladder Cancer Tissues and Tumor-Adjacent Normal Tissues

**Figure 2:**
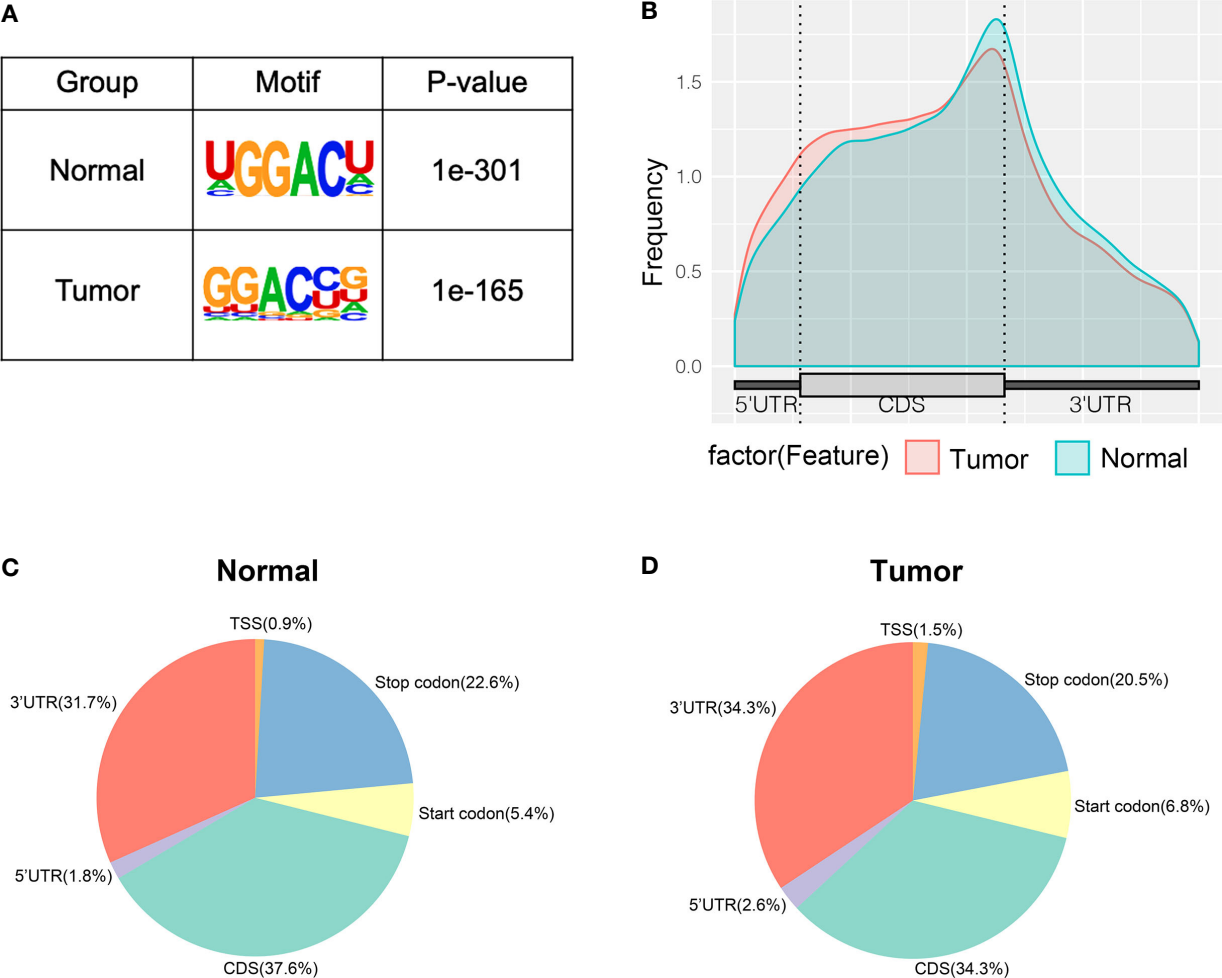
Characteristics of m^6^A peaks within mRNAs, lncRNAs, and circRNAs in bladder cancer tissues and adjacent normal tissues. **(A)** Sequence motif of m^6^A-containing peak regions in tumor and adjacent normal tissues respectively. **(B)** The metagene profiles of transcripts peaks in tumor and adjacent normal tissues. **(C, D)** The proportion of m^6^A peaks in the whole transcriptome of tumor and adjacent normal tissues.

To study whether the m^6^A peaks recognized by us had conserved the RRACH motif, we performed the HOMER motif software to analyze the m^6^A peaks that we identified from the MeRIP-seq data. In the normal and tumor groups, the motif sequence was GGACU and GGACC, respectively (**Figure 2A**). This showed that there was a difference of m^6^A motif in tumor and adjacent normal tissues, but their motif sequences were similar to those previously identified. To make clear the priority position of m^6^A in the whole transcriptome of bladder cancer tissues and adjacent normal tissues, we then studied the metagene profiles of transcript peaks in the two groups. We observed that the m^6^A peaks were mostly located at the end of the 5′UTRs and start of the 3′UTRs in tumor tissues and adjacent normal tissues (**Figure 2B**). In addition, we found that the proportion of m^6^A peaks located at CDS was the highest and the proportion of m^6^A peaks located at TSS was the least in both tissues (**Figures 2C, D**).

**Figure 3:**
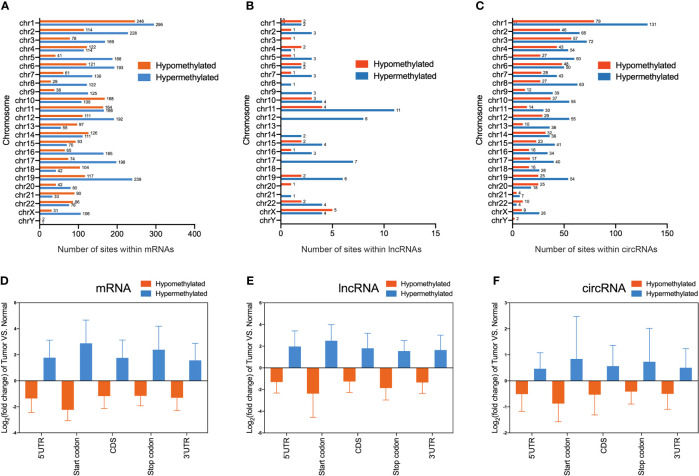
Distribution of differentially methylated N^6^-methyladenosine sites between bladder cancer tissues and adjacent normal tissues. **(A–C)** Chromosomal distribution of all differentially methylated N^6^-methyladenosine sites within mRNAs **(A)**, lncRNAs **(B)**, and circRNAs **(C)**. **(D–F)** Statistics of fold change of differentially methylated N^6^-methyladenosine peaks within mRNAs **(D)**, lncRNAs **(E)**, and circRNAs **(F)** in five segments.

To obtain the distribution profiles of all differentially m^6^A methylated mRNAs, lncRNAs, and circRNAs across chromosomes, the containment of differentially methylated m^6^A sites harbored by chromosomes was classified by respective chromosome. This result showed that hypermethylated and hypomethylated m^6^A sites within mRNAs were primarily located on chromosomes 1, 2, and 19 (**Figure 3A**). Hypermethylated and hypomethylated m^6^A sites within lncRNAs were primarily located on chromosomes 11, 12, and X (**Figure 3B**). Moreover, hypermethylated and hypomethylated m^6^A sites within circRNAs were primarily located on chromosomes 1, 2, and 3 (**Figure 3C**). Totally, the top three chromosomes containing the differentially methylated m^6^A sites were chromosomes 1, 2, and 19. Then, these hypermethylated and hypomethylated m^6^A sites within mRNAs, lncRNAs, and circRNAs were classified by five regions. For both hypermethylated and hypomethylated mRNAs, lncRNAs, and circRNAs, the fold change of the start codon region was the highest (**Figures 3D–F**). These results of the distribution of m^6^A modifications were similar to those of previous studies.

### Functional Analysis of Differentially m^6^A Methylated mRNAs, lncRNAs, and circRNAs Between Two Groups

**Figure 4:**
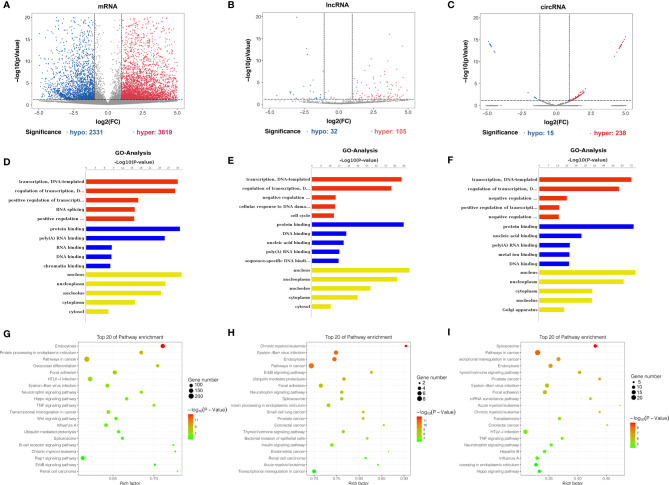
Gene ontology and KEGG pathway analyses of genes harboring differentially methylated N6-methyladenosine sites. **(A–C)** Volcano plots showing differentially m^6^A-modified mRNAs **(A)**, lncRNAs **(B)**, and circRNAs **(C)** based on |log2FC| > 1 and p-value < 0.05. In volcano plots, red blots represent hypermethylation and blue blots represent hypomethylation. **(D–F)** Gene ontology functional enrichment analysis results of differentially methylated m^6^A sites-contained mRNAs **(D)**, lncRNAs **(E)** and circRNAs-associated **(F)** genes. **(G–I)** KEGG pathway analysis results of differentially methylated m^6^A sites-contained mRNAs **(G)**, lncRNAs **(H)**, and circRNAs-associated genes **(I)**.

Differentially m^6^A methylated mRNAs, lncRNAs, and circRNAs were identified between bladder cancer tissues and adjacent normal tissues based on |log2FC| > 1 and p-value < 0.05. Totally, volcano plots showed 2,331 hypomethylated and 3,819 hypermethylated mRNAs (**Figure 4A**), 32 hypomethylated and 105 hypermethylated lncRNAs (**Figure 4B**), and 15 hypomethylated and 238 hypermethylated circRNAs (**Figure 4C**) in bladder cancer tissues compared to adjacent normal tissues. To uncover the functions of m^6^A methylation modification in bladder cancer, differentially methylated mRNAs, lncRNAs, and circRNAs between tissues were selected for Gene Ontology enrichment analysis and Kyoto Encyclopedia of Genes and Genomes pathway analysis. The results of GO analysis showed that differentially m^6^A methylated mRNAs were mostly enriched in regulation of transcription and RNA splicing (**Figure 4D**), differentially m^6^A methylated lncRNAs were mostly enriched in protein binding and cell cycle (**Figure 4E**), and differentially m^6^A methylated circRNAs were mostly enriched in the transcription process and nucleic acid binding (**Figure 4F**). Furthermore, KEGG pathway analysis showed that differentially m^6^A methylated mRNAs were mostly involved in TNF signaling pathway and transcriptional misregulation in cancer (**Figure 4G**), differentially m^6^A methylated lncRNAs were mostly enriched in pathways in cancer and endocytosis (**Figure 4H**), and differentially m^6^A methylated circRNAs were mostly enriched in spliceosome and mRNA surveillance pathway (**Figure 4I**). In summary, we found that differentially m^6^A methylated genes identified from bladder cancer tissues were involved in important biological processes and pathways.

### Conjoint Analysis of MeRIP-seq and RNA-seq Results Between Two Groups

**Figure 5:**
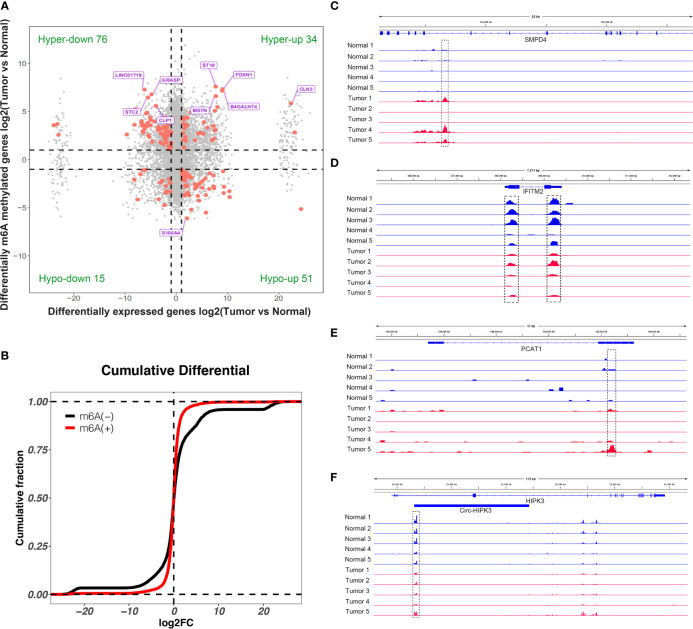
Conjoint analysis of differentially methylated genes and differentially expressed genes. **(A)** Four-quadrant diagram showed the differentially methylated genes and differentially expressed genes in tumor and adjacent normal tissues. **(B)** Cumulative distribution of gene expression between tumor and adjacent normal tissues for m^6^A transcripts (red) and non-m6A transcripts (black). **(C–F)** Integrative Genome Viewer (IGV) software showed representative differentially methylated mRNAs (SMPD4 and IFITM2), lncRNA (PCAT1), and circRNA (circ-HIPK3) in tumor and adjacent normal tissues.

By conjoint analysis of the results from MeRIP-seq and RNA-seq between tissues, we found that there were 34 hypermethylated and upregulated (hyper-up) genes, 15 hypomethylated and downregulated (hypo-down) genes, 76 hypermethylated and downregulated (hyper-down) genes, and 51 hypomethylated and upregulated (hypo-up) genes in bladder cancer tissues compared to adjacent normal tissues (**Figure 5A**). To further analyze whether m6A methylation affects gene expression, we divided all expressed transcripts into m^6^A transcripts and non-m^6^A transcripts, calculated the log two-fold change (log2FC) values of these transcripts, and generated a cumulative curve. The result revealed that the proportion of transcripts modified by m^6^A was larger than that of transcripts not modified by m6A, especially in terms of the log2FC of the transcript FPKM value between 0 and 20 (**Figure 5B**). This result promoted us to investigate the general locations of differentially methylated m^6^A sites within bladder cancer- or other tumor-related genes in bladder cancer tissues compared to adjacent normal tissues. For instance, sphingomyelin phosphodiesterase 4 (SMPD4) was overexpressed in the late stage of clear cell renal cancer and acted as a biomarker for discriminating the early and late stages of ccRCC (22). We found that the m^6^A peak was enriched around the 5′UTR of SMPD4 in the tumor group of bladder cancer not in adjacent normal tissues (**Figure 5C**). Moreover, interferon-induced transmembrane protein 2 (IFITM2) promotes gastric cancer growth and metastasis (23), within which m^6^A was hypomethylated (bladder cancer tissues vs. normal adjacent tissues) and enriched in coding sequence (CDS) (**Figure 5D**). Within lncRNA PCAT1, a significantly hypermethylated m^6^A peak enriched in exon 2 was shown in tumor tissues (**Figure 5E**) and has been reported to suppress castration-resistant prostate cancer progression by activating AKT and NF-κB signaling (24). Circular RNA circ-HIPK3 is downregulated and suppresses cell proliferation, migration, and invasion in osteosarcoma (25) and shows a significantly hypomethylated m^6^A peak in the tumor group (**Figure 5F**).

### Expression of Candidate Genes Correlates With Worse Overall Survival in Bladder Cancer Patients

**Figure 6:**
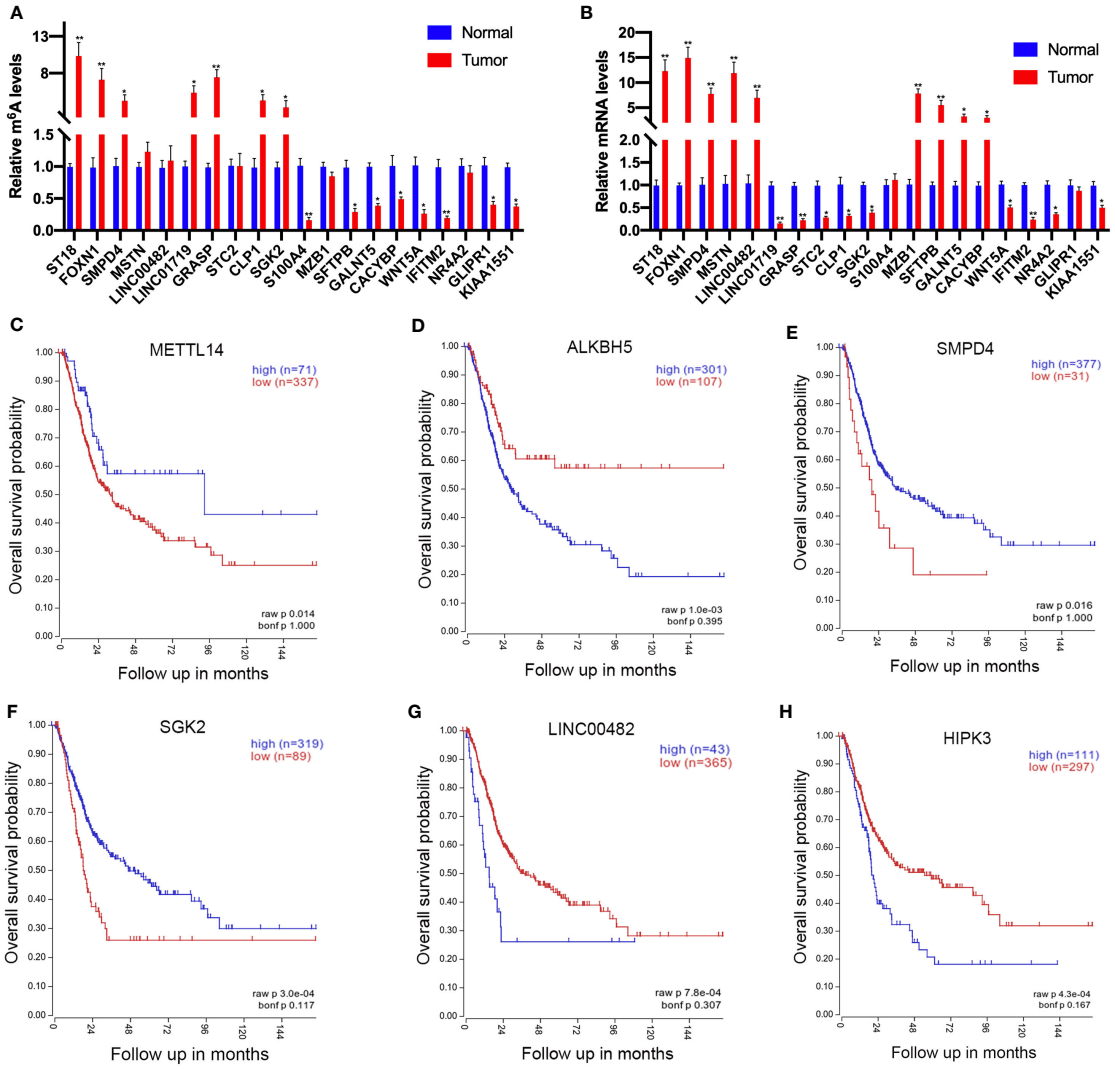
Prognostic value of the survival-associated gene signature in bladder cancer patients. **(A)** Validations of the m^6^A enrichments of 10 hypermethylated genes and 10 hypomethylated genes by m^6^A-immunoprecipitation (IP)-qPCR. **(B)** Validations of the mRNA expression levels of 10 upregulated genes and 10 downregulated genes by RT-qPCR. **(C–H)** The low expressions of METTL14 **(C)**, SMPD4 **(E)**, and SGK2 **(F)** in mRNA level correlate with worse overall survival in bladder cancer patients. The high expression of ALKBH5 **(D)**, LINC00482 **(G)**, and HIPK **(H)** expression levels showed a tendency to correlate with worse overall survival in bladder cancer patients. *p < 0.05, **p < 0.01.

To further confirm the results of our m^6^A-seq data, we conducted gene-specific m^6^A-IP qPCR assays for 10 hypermethylated (ST18, FOXN1, SMPD4, MSTN, LINC00482, LINC01719, GRASP, STC2, CLP1, and SGK2) and 10 hypomethylated genes (S100A4, MZB1, SFTPB, GALNT5, CACYBP, WNT5A, PRR16, NR4A2, GLIPR1, and KIAA1551) which might participate in tumor progression in bladder cancer. We observed the almost same m^6^A-level changes in these genes, confirming the validity of our MeRIP-seq results (**Figure 6A**). Sequentially, transcript levels of the abovementioned genes (ST18, FOXN1, SMPD4, MSTN, LINC00482, S100A4, MZB1, SFTPB, GALNT5, and CACYBP were upregulated genes, LINC01719, GRASP, STC2, CLP1, SGK2, WNT5A, PRR16, NR4A2, GLIPR1, and KIAA1551 were downregulated genes) were also measured in five pairs of bladder cancer and adjacent normal tissues by RT-qPCR (**Figure 6B**). Results showed a similar tendency of transcript levels with RNA-seq data in two groups, which validated our RNA-seq results. To confirm the clinical significance of the candidate genes discovered in this study, Kaplan–Meier analysis extracted from the TCGA database was explored. We found that a low expression of METTL14 (a m6A methyltransferase), SMPD4, and SGK2, but a high expression of ALKBH5 (a m6A de methyltransferase), LINC00482, and HIPK3, showed a tendency to associate with worse overall survival in bladder cancer patients (**Figures 6C–H**).

## Discussion

N6-methyladenosine (m^6^A) is the most common mRNA modification in eukaryotic cells of all higher animals (26). It is involved in various physiological and pathological processes by regulating mRNA transcription, processing, and other metabolic processes (27–32). At present, MeRIP-seq was used to study the distribution sites and expression levels of m^6^A on transcripts in mammalian cells, and it was found that m^6^A was distributed in the entire transcriptome including mRNA and non-coding RNA, mainly concentrated in the 3′-UTR and near the transcriptional stop codon (14). Studies have shown that m^6^A is dynamically regulated by methyltransferase and demethyltransferase, but the biological function of m^6^A in cancer is not yet fully understood (33). In this study, bladder cancer tissues and normal adjacent tissues were created to assess the m^6^A state, which revealed big differences between the tumor and adjacent normal groups, supporting the dynamic characteristic of m^6^A modification.

In the current study, we figured out that m^6^A modification in tumor and adjacent normal tissues mainly occurs in the motif, GGACC and GGACU, respectively, which is similar to the previous data. Moreover, transcript methylated peaks are mainly located at CDS. Almost 85% of methylated genes have one to five m^6^A methylated sites, and others contain over five m^6^A methylated sites in mRNAs, lncRNAs, and circRNAs of tumor and adjacent normal tissues. In addition, differentially methylated genes between tumor and adjacent normal tissues were detected and shown to be involved in many important biological pathways such as pathways in cancer, transcriptional misregulation in cancer, TNF signaling pathway, and hippo signaling pathway. Studies have reported that TNF-alpha induced MMP-9 expression in bladder cancer cells by activating the transcription factor NF-kappaB, which is involved in the p38 MAP kinase-mediated control of MMP-9 regulation (34). Another study reported that the Hippo signaling pathway is a conserved pathway that plays a crucial role in cellular proliferation, differentiation, and apoptosis in bladder cancer (35). A combined analysis of our MeRIP-seq and mRNA-seq data revealed 34 hyper-up genes, 15 hypo-down genes, 76 hyper-down genes, and 51 hypo-up genes in the tumor group compared with the adjacent normal group; these genes may play critical roles in the development of bladder cancer. Moreover, some of these genes were reported to facilitate tumor growth and metastasis in different kinds of cancers. For example, SMPD4 was overexpressed in the late stage of clear cell renal cancer and acted as a biomarker for discriminating early and late stages of ccRCC (22), but its function in bladder cancer is unclear. In this study, we found that SMPD4 was hypermethylated and upregulated in bladder cancer tissues in comparison to normal adjacent tissues, and a low expression of SMPD4 showed a tendency to associate with worse overall survival in bladder cancer patients. Our data indicate that m^6^A methylation could participate in tumor progression through the modification of tumor-related genes. However, further experiments should be required to confirm these results.

m^6^A modification is involved in almost every step in mRNA metabolism. Furthermore, it also affects the processing of lncRNAs and circRNAs. Our findings provide the first m^6^A modification landscape in bladder cancer. Differentially expressed mRNAs with hyper-methylated or hypo-methylated m^6^A modifications are identified, which may help observe the mechanisms of m^6^A-mediated gene expression regulation. In further studies, we will evaluate the biological relevance and clinical value of m^6^A in bladder cancer.

## Materials and Methods

### Patients and Samples

Five pairs of bladder cancer tissues and adjacent non-malignant tissues with patients’ informed consent were obtained from the Urology Department of Peking University First Hospital (PKUFH), Beijing, China. This study followed the Helsinki declaration and was approved by the Institutional Ethical Review Board of PKUFH. Samples were collected immediately in the operating room after surgical removal and were stored in liquid nitrogen after rapid freezing in liquid nitrogen for the following RNA isolation.

### MeRIP-seq and RNA-seq of the Whole Transcriptome

MeRIP-seq and RNA-seq were performed at CloudSeq Biotech, Inc. (Shanghai, China) (36–38) and as described previously. Briefly, total RNAs were isolated from five pairs of bladder cancer tissues and normal adjacent tissues using TRIzol (Invitrogen). Then, total RNA was fragmented into almost 100 nt and were incubated with anti-m^6^A antibody (Manga) for 2 h at 4°C. Then, the beads were prepared and incubated with the total RNA for 2 h at 4°C. Finally, the mixture was washed and the m^6^A-bound RNA was purified with TE buffer. After being purified, the samples were used to construct the library by Prep Kit (Illumina) on HiSeq 3000.

### MeRIP-seq and Data Analysis

Total RNA was extracted from the two groups of cells by using TRIzol Reagent (Life Technologies). The quality and quantity of total RNA were assessed by using NanoDrop ND 2000 (Thermo Fisher Scientific, MA, USA). The RNA integrity was measured using denaturing agarose. Seq-Star T M poly(A) mRNA Isolation Kit (Arraystar, MD, USA) was used to isolate mRNA from total RNA. The GenSeq™ m6A RNA IP Kit (GenSeq Inc., China) was used to perform m6A RNA immunoprecipitation by following the manufacturer’s instructions. The input samples without immunoprecipitation and the m6A IP samples were both used for library construction with NEBNext Ultra II Directional RNA Library Prep Kit (New England Biolabs, Inc., MA, USA). The library quality was evaluated with the Bioanalyzer 2100 system (Agilent Technologies Inc., CA, USA). Library sequencing was performed on an Illumina HiSeq instrument with 150-bp paired-end reads.

Paired-end reads were harvested using the Illumina HiSeq 4000 sequencer and were quality controlled by Q30. After 3′ adaptor-trimming, low-quality reads were removed by Cutadapt software (v1.9.3). First, clean reads of all libraries were aligned to the reference genome (HG19) by Hisat2 software (v2.0.4). Methylated sites on RNAs (peaks) were identified by MACS software (39). Identified m6A peaks were subjected to motif enrichment analysis by HOMER (40), and metagene m6A distribution was characterized by R package MetaPlotR (41). Differentially methylated sites (fold change ≥2 and p < 0.05) were identified by diffReps (42). These peaks identified by software overlapping with exons of mRNA were figured out and chosen by homemade scripts. Genes of interest were visualized in the IGV (Integrative Genomics Viewer) software (v2.3.68) (43). The gene ontology (GO) analysis and pathway enrichment analysis were performed on the differentially methylated protein-coding genes by using the GO (www.geneontology.org) and Kyoto Encyclopedia of Genes and Genomes (KEGG) databases (www.genome.jp/kegg). Clinical survival data (including expression level and survival time) were downloaded from the TCGA database (https://cancergenome.nih.gov/).

### RNA-seq and Data Analysis

Total RNA was extracted from biological samples using TRIzol Reagent (Life Technologies) according to the manufacturer’s protocol. Denatured agarose gel electrophoresis was applied to evaluate the integrity of total RNA. Seq-Star TM poly(A) mRNA Isolation Kit (Arraystar, MD, USA) was used to purify mRNA from total RNA after confirming its quantity and quality by NanoDrop ND-2000. Then, fragmented mRNA was subjected to 50-bp single-end sequencing with a BGISEQ-500 platform. Adapter and low-quality reads were trimmed by SOAPnuke (44), and trimmed reads were aligned to reference genome by bowtie2 (45). RSEM (46) was used to calculate expression levels, and DEGs were identified by DEGseq (47).

### M^6^A-IP-qPCR and RT-qPCR

Twenty genes with differentially methylated sites according to MeRIP-seq were tested by reverse transcription (RT)-qPCR. A small number of fragmented RNA was used as the input control. The rested RNA was incubated with anti-m6A antibody-coupled beads. The m6A-containing RNAs were then immunoprecipitated and eluted from the beads. Both input control and m^6^A-IP samples were subjected to RT-qPCR with gene-specific primers.

### Statistical Analysis

Experiments were performed at least three times, and representative results are shown. All statistical analyses were performed and visualized using RStudio (Version1.2.1335, Boston, MA, USA), GSEA (Version4.0, UC San Diego and Broad Institute, USA) 23, MedCalc (Version16.8, Ostend, Belgium), and GraphPad Prism (Version 8.0, GraphPad, Inc., La Jolla, CA, USA). Differences between individual groups were analyzed using the chi-squared test and Student’s t-test (two-tailed and unpaired) with triplicate or quadruplicate sets. A two-tailed p < 0.05 was considered statistically significant.

## Data Availability Statement

All RNA sequencing data were deposited in the NCBI SRA (Sequence Read Achieve) database with the accession number of PRJNA733602.

## Ethics Statement

The studies involving human participants were reviewed and approved by the Institutional Ethical Review Board of PKUFH. The patients/participants provided their written informed consent to participate in this study. Written informed consent was obtained from the individual(s) for the publication of any potentially identifiable images or data included in this article.

## Author Contributions

AL, YG, and CC performed the experiments and data analysis. CC and QuZ prepared the diagrams and wrote the manuscript. AL and YG designed the project. QiZ and LY supervised the project and provided financial support. All authors contributed to the article and approved the submitted version.

## Funding

This work was supported by grants from the National Natural Science Foundation of China to QZ (Nos. 82072826 and 81872088) and a grant from Peking University Medicine Fund of Fostering Young Scholars’ Scientific & Technological Innovation to YG (No. BMU2020PYB028).

## Conflict of Interest

The authors declare that the research was conducted in the absence of any commercial or financial relationships that could be construed as a potential conflict of interest.

## Publisher’s Note

All claims expressed in this article are solely those of the authors and do not necessarily represent those of their affiliated organizations, or those of the publisher, the editors and the reviewers. Any product that may be evaluated in this article, or claim that may be made by its manufacturer, is not guaranteed or endorsed by the publisher.
